# A View from Inside and Outside the Tree: Using Different Techniques to Assess the Use of Black Woodpecker Cavities in Forests

**DOI:** 10.3390/biology15191687

**Published:** 2026-09-22

**Authors:** Grzegorz Zawadzki, Dorota Zawadzka

**Affiliations:** 1 Department of Silviculture and Forest Protection, Warsaw University of Life Sciences, Nowoursynowska 159, 02-776 Warsaw, Poland; grzegorz_zawadzki@sggw.edu.pl; 2 Department of Forest Sciences, Branch in Tomaszów Mazowiecki, University of Łódź, Konstytucji 3 Maja 65/67, 97-200 Tomaszów Mazowiecki, Poland

**Keywords:** *Dryocopus martius*, Black Woodpecker cavity, secondary cavity nesters, Hymenoptera, camera traps

## Abstract

Large cavities are important for maintaining forest biodiversity. However, accurately assessing cavity use and identifying the communities of organisms associated with these structures remain challenging and are still insufficiently studied. Between 2022 and 2026, tree cavities excavated by the Black Woodpecker (*Dryocopus martius*) in northeastern Poland were monitored using two complementary techniques: camera traps installed near cavity entrances and an action camera used to inspect cavity interiors. The aim of this study was to assess the diversity of animal communities associated with Black Woodpecker cavities and to compare the effectiveness of the two monitoring methods. In total, 32 bird species, six mammal species, and 11 insect species were recorded as using Black Woodpecker cavities. Inspections of cavity interiors confirmed breeding by 19 bird species and 4 mammal species, and 4 insect species. Camera traps recorded 29 bird species, four mammal species, and one species of social insect at cavity entrances. Over half (51.7%) of the bird species recorded at cavity entrances were not detected inside the cavities, whereas 17.6% of bird species confirmed to breed in the cavities were not detected by camera traps. Camera traps also did not allow for the identification of bat species or the detection of insects other than hornets. The results demonstrate that the assemblages of animals associated with Black Woodpecker cavities differed depending on the monitoring method used. Traditional observation methods may underestimate the diversity of cavity-associated fauna, particularly species that remain hidden inside cavities or use them only during specific stages of their life cycle. The two modern techniques used in this study provided complementary information and should therefore be considered together when assessing the diversity and use of tree cavities by forest fauna.

## Simple Summary

Tree cavities are important ecological structures in forest ecosystems. In the Augustów Forest, northeastern Poland, we investigated the importance of cavities excavated by the Black Woodpecker (*Dryocopus martius*) for forest-dwelling animals. We used two complementary methods: (1) 33 camera traps installed near cavity entrances on neighboring trees and (2) inspections of 681 cavities using a camera mounted on a telescopic pole to examine the interior of the cavity chamber. In total, we confirmed the presence of 50 animal species associated with the studied cavities. Birds were the most diverse group (32 species), followed by insects (11 species) and mammals (6 species). Among the recorded taxa, at least 19 bird species, four mammal species, and four insect species bred in the cavities. Other species visited and explored the cavities, using them for resting, feeding, or foraging. The results obtained using the two camera-based methods differed in the type of information provided. Camera traps recorded animals appearing at cavity entrances over extended periods, whereas cameras inside cavity chamber provided a snapshot of cavity contents at the time of inspection, including evidence of reproductive activity. Thus, the two methods provided complementary information on cavity use by forest animals. Camera-trap recordings revealed 34 animal species, of which 85% were birds, 12% mammals, and 3% insects. The most frequently recorded species was the Black Woodpecker, followed by the Stock Dove (*Columba oenas*), Great Tit (*Parus major*), Eurasian Pygmy Owl (*Glaucidium passerinum*), and Great Spotted Woodpecker (*Dendrocopos major*). The Common Goldeneye (*Bucephala clangula*), Eurasian Nuthatch (*Sitta europaea*), and Beech Marten (*Martes foina*) were recorded at more than 20% of the studied cavities. During cavity inspections, the presence of at least 33 taxa was confirmed. Of these, 52% were birds, 15% mammals, and 33% invertebrates. The most frequently observed species was the Stock Dove, followed by the Black Woodpecker, Common Goldeneye, and European Hornet (*Vespa crabro*). Our results confirm the important role of large tree cavities in maintaining forest biodiversity. They also highlight the complex network of relationships between cavities excavated by the Black Woodpecker and forest fauna, demonstrating that these structures may serve as important sites for reproduction, shelter, resting, and foraging.

## 1. Introduction

Tree cavities are used by a wide range of animals, including small- and medium-sized vertebrates as well as invertebrates. Cavities provide breeding sites and shelters for hiding and resting [1,2,3,4]. Approximately 10–40% of bird and mammal species worldwide use tree cavities for breeding or resting [1]. In Central Europe, cavity-dependent vertebrates include approximately 40 bird species and at least 20 mammal species [5]. Non-breeding uses of cavities include exploration for feeding and food storage [6,7]. Thus, tree cavities are considered keystone structures for maintaining forest biodiversity [8,9,10].

Not all animal species can use all types of tree cavities. The dimensions of the cavity entrance determine whether a cavity can be accessed and used by a particular species, depending largely on body size. In general, larger animals can use only large cavities, whereas smaller animals can access cavities of a wider range of sizes. Consequently, large tree cavities, with entrance dimensions of at least 10 cm, represent a particularly valuable resource because they are relatively rare in forest ecosystems. In conifer-dominated and mixed forests of Central Europe, such cavities are created primarily by the Black Woodpecker (*Dryocopus martius*) [4,11]. These cavities can be used by medium-sized birds and mammals [6,12,13]. Large tree cavities are also important for social insects that construct large nests, including the Honey Bee (*Apis mellifera*) and the European Hornet (*Vespa crabro*) [5,14,15].

Many studies have highlighted the importance of tree cavities, particularly large cavities, for the conservation of biodiversity in forest ecosystems [8,16,17,18]. However, accurately assessing cavity use and identifying the organisms associated with these structures remain challenging and are still insufficiently studied. Data on the species composition of animal assemblages using tree cavities across different regions are scarce. This is largely due to the difficulty of inspecting cavity contents, as cavities are often located at considerable heights above the ground. Until recently, the only methods available for inspecting the interior of tree cavities involved using a ladder or climbing the tree, both of which are physically demanding and time-consuming. Examination of the concealed cavity chamber often required the use of a mirror [19,20]. Based on direct inspections of Black Woodpecker cavities in Sweden, Johnsson et al. [16] documented the use of these cavities by 14 animal species, including birds, mammals, and social insects.

New technologies have facilitated the monitoring of tree cavity use. One such technology is the camera trap, which has been widely used to monitor terrestrial mammal populations. For mammals and ground-dwelling birds, camera traps are particularly useful for species inventories, occupancy studies, analyses of activity patterns and behavior, and long-term monitoring [21,22]. The use of camera traps in vertebrate research has become increasingly important because they provide information that was previously difficult or impossible to obtain [23,24]. Although camera traps are increasingly used on a large scale, their application has focused primarily on mammals [25,26,27,28]. Camera traps installed close to the ground can be relatively easily deployed and monitored, whereas their use in trees presents additional logistical challenges. Nevertheless, given the inaccessibility of tree cavities and canopies, camera traps may provide valuable information on the ecology and behavior of cavity-associated animals [29,30]. To date, only a limited number of studies have used camera traps positioned near tree cavities [31,32,33]. In Europe, such studies have been conducted in large cavities excavated by woodpeckers [34] and in large decay-formed cavities [6], providing information on the species composition of animal assemblages visiting and using tree cavities, as well as on interactions among cavity-associated species.

Another relatively recent approach involves the use of a video camera mounted on a fiberglass pole. This technique enables the interior of a cavity to be inspected from the ground [35,36]. The camera can be positioned inside the cavity chamber and used to record its contents as video or still images. This approach provides direct information on the conditions and organisms present inside a cavity at the time of inspection [35,37]. To date, only a limited number of studies have used pole-mounted cameras to monitor tree cavities, and most have been conducted outside Europe [38].

The aim of this study was to assess the diversity of animal species using cavities excavated by the Black Woodpecker, a bird species that creates the largest cavities in European forests [12,13]. We used two complementary technologies: camera traps positioned near cavity entrances and camera-based inspections of cavity interiors. These methods were used to identify the species composition of animal assemblages associated with Black Woodpecker cavities and to characterize the ways in which cavities were used.

We compared the results obtained using the two methods and assessed the extent to which they provided complementary information on cavity use. We hypothesized that Black Woodpecker cavities play the greatest role in the reproduction of medium-sized birds, whereas their importance for smaller bird species is relatively limited. We also expected that camera traps positioned near cavity entrances would detect a greater number of species associated with tree cavities than cameras used to inspect cavity interiors.

## 2. Materials and Methods

### 2.1. Study Area

The Augustów Forest is located in northeast Poland (23°15′ E, 53°54′ N) and covers over 1400 km^2^. The area is relatively flat, with elevations between 135 and 190 m a.s.l. The climate is rather cold, with a mean annual temperature of 6.5 °C [39]. Forest cover is approximately 90% of the area, while lakes account for 6%. The Augustów Forest lies in the basin of four rivers and includes 25 smaller rivers and streams, as well as 15 canals. Tree stands are dominated by the Scots pine (*Pinus sylvestris*) (77.5%). The Norway spruce (*Picea abies*) accounts for 8% and occurs across most forest site types, the black alder (*Alnus glutinosa*) (8.5%) grows mainly on wet sites, the silver birch (*Betula pendula*) (5%) occurs mainly on mesic sites, and pedunculate oak (*Ouercus robur*) covers approximately 1% of the stands. A substantial part of the forest consists of even-aged forests, with trees of similar age formed by one dominant species with admixtures of several others. The mean stand age is 65 years, and stands older than 100 years comprise approximately 15% of the forest area [40]. Among the forest site types, mesic pine forests cover nearly 40% of the area, while mesic mixed forests account for a further 27%. Wet forest types constitute approximately 7% of the forested area. The Augustów Forest is included in the Natura 2000 network as a Special Protection Area for Birds (PLB200002 “Puszcza Augustowska”, English Augustów Forest). Nearly 90% of the area consists of commercial stands managed by six forest districts of the State Forests National Forest Holding. In these areas, intensive timber harvesting, including clear-cutting, is practiced, and trees weakened or attacked by insects or fungi are routinely removed. The Wigry National Park covers 150 km^2^ and is located in the northwestern part of the Augustów Forest. There are no clearings, and trees weakened by insects or fungi are removed only when they cause a risk to public safety [41].

### 2.2. Field Data Collection

From 2022 to 2026, a study of the use of Black Woodpecker cavities was conducted in the Augustów Forest. The cavities inspected during the study had been identified in previous years as part of research on the ecology of the Black Woodpecker. The necessary permits for conducting the research were obtained from the Ministry of Climate and Environment (DOP-WPN.61.37.2022.DW) and the Regional Directorate for Environmental Protection in Białystok (RDOŚ) WPN.6401.110.2023.DO).

Two complementary techniques were used to monitor animals using Black Woodpecker cavities. The first involved installing camera traps on a neighboring tree directly opposite the cavity entrance. Three camera-trap models were used: SpecOps (Browning, Birmingham, AL, USA), SP1390 (Bentech EU, Prague, Czech Republic), and Spromise S68 (Tetrao, Bratislava, Slovakia). In total, camera traps were installed at 33 trees with cavities between 2022 and 2024. The camera traps were installed in early April, at the beginning of the breeding season, and remained in the field until autumn, collecting data for as long as the batteries lasted. The cameras were set to motion-detection mode and recorded photographs and videos in response to detected movement. This approach enabled continuous monitoring of animals appearing at or near cavity entrances. The batteries were not replaced during the monitoring period, and the camera traps were reinstalled at the selected cavities in the following season.

Camera traps were installed only at cavities for which a suitable neighboring tree was available to position the camera directly opposite the entrance. In 2022, cavities were selected when a neighboring tree suitable for camera installation was located within a radius of up to 8 m from the cavity entrance. In subsequent years, efforts were made to select cavities with a suitable mounting tree located no more than 6 m from the cavity. Camera installation was performed by a professional tree climber and was possible only on living trees for safety reasons. A total of 60% of the camera traps were installed in the national park, but only 15% of the surveyed tree cavities were located there.

Because this method provided information primarily on animals appearing outside the cavity and did not allow for their actual use of the cavity to be determined reliably, a second method was employed to directly inspect the cavity chamber. The second method involved using an action camera mounted on a telescopic pole to assess cavity contents and determine actual cavity use. Two camera models were used during the study: Action 2 (DJI, Shenzhen, China) and GoPro HERO11 Black Mini (GoPro, San Mateo, CA, USA). A flashlight attached to the camera was used to illuminate the cavity interior. Approximately 200 unique Black Woodpecker cavities were initially selected for monitoring using this method. In the subsequent years (2022–2026), 39, 140, 168, 186, and 148 cavities were inspected, respectively, with an average of 136 cavities inspected per year. In 2026, the research was conducted through August. Variation in sample size among years resulted from environmental changes, cavity availability, and the disappearance of cavities due to natural and anthropogenic causes [42]. The statistical unit for further analysis was a single tree cavity, monitored for one to five seasons. When a specific tree cavity was occupied more than once by the same species, the occupation was not counted again to avoid pseudoreplication. Each season was treated separately, and the results obtained in each year were then averaged to determine the proportion of tree cavities occupied by individual species.

Inspections began in late March, at the beginning of the breeding season. The telescopic pole was 14 m long and, together with the height of the operator, allowed for cavities located up to approximately 15 m above the ground to be inspected. This covered more than 78% of the cavities identified in the study area [43]. Subsequent inspections were conducted at approximately 3–4-week intervals to monitor breeding activity and identify species using the cavities before the completion of breeding and departure from the cavities. During each inspection, a video recording was made to document the contents of the cavity. The camera remained inside the cavity for no longer than 1 min, while individual recordings typically lasted 20–30 s. The complete inspection of a single cavity took approximately 3–5 min.

### 2.3. Data Analysis

All photographs and video recordings obtained using camera traps and the pole-mounted camera were subsequently analyzed on a computer using a 24-inch high-resolution monitor. Organisms were identified to the highest taxonomic resolution possible. All birds were identified to the species level, as were most mammals, whereas insects were generally identified to higher taxonomic levels, primarily genus or family.

For camera-trap recordings, all organisms appearing at the cavity entrance or within the entrance area were recorded. Behavioral patterns were not analyzed because individual photographs often did not provide sufficient evidence to determine whether an animal had actually entered the cavity. Animals appearing only in the wider vicinity of the cavity or on tree branches visible in the background of the camera-trap frame were excluded from the analysis.

Recordings obtained using the pole-mounted camera were analyzed in their entirety to identify organisms present inside the cavity. Taxa were identified to the species level whenever possible or otherwise assigned to a higher taxonomic level. All observations of organisms present inside the cavity were recorded. The complete dataset was compiled in a Microsoft Excel database. The raw data used in further analyses are included in the Supplementary Materials.

For both datasets, the Shannon–Wiener diversity index [44] and an occurrence index, expressed as the percentage of cavities in which a given taxon was recorded, were calculated for each taxon. Data from all study seasons were pooled to provide an overall assessment of the animal assemblages associated with Black Woodpecker cavities.

The species composition of birds and the taxonomic composition of all recorded organisms were then compared between the two monitoring methods. To characterize the overall importance of Black Woodpecker cavities for biodiversity, records obtained using both methods were combined to create a single list of taxa associated with the cavities.

Classification of cavity use

Animal records were assigned to four categories of cavity use:

- Breeding—use of the cavity for reproduction and raising offspring;
- Resting—use of the cavity as a shelter or hiding place;
- Visiting—temporary exploration or inspection of the cavity;
- Feeding—use of the cavity for foraging, feeding, or collecting food resources.

Categories were assigned based on the observed behavior of animals associated with the cavities. Frequent and regular visits, changes in partners within the cavity, food provisioning, the presence of young at the cavity entrance, or the detection of offspring inside the cavity were considered evidence of breeding. In contrast, irregular appearances outside the breeding season (July–September), particularly when not preceded by previous observations of the species, were classified as resting. Individual observations of birds inside a cavity that were not repeated during subsequent surveys were classified as visiting. Observations indicating foraging, removal of cavity contents, or feeding inside the cavity were assigned to the feeding category. Isolated incidental photographs for which no specific type of cavity use could be established were also classified as visiting.

Classification according to intensity of cavity use

Species were also classified according to the frequency with which they were recorded at the surveyed cavities. The difference in the thresholds used for both methods stems from an assessment of the potential frequency of data collection. While the camera traps operated continuously for 2–5 months, the pole-based surveys provided data from 4–7 points in time during the season. Therefore, it is not possible to obtain comparable occurrence frequencies of species using the two methods, a fact that was taken into account when establishing the thresholds. Three categories of cavity use intensity were distinguished:

- Keystone user—cavities represented the primary or exclusive type of cavity resource used by a specialized species, with the species recorded in more than 20% of cavities monitored using camera traps or more than 10% of cavities inspected using the pole-mounted camera;
- Facultative user—cavities represented one of several types of breeding or shelter sites used by a species, with the species recorded in more than 10% and less than 20% of cavities monitored using camera traps or more than 3% and less than 10% of cavities inspected using the pole-mounted camera;
- Accidental user—cavities were used only sporadically, with the species recorded in less than 10% of cavities monitored using camera traps or less than 3% of cavities inspected using the pole-mounted camera.

### 2.4. Statistical Analyses

The Kruskal–Wallis test was used to compare the frequency of cavity use among taxa belonging to different taxonomic classes, followed by Dunn’s test for post hoc pairwise comparisons. The Wilcoxon signed-rank test was used to compare the proportions of taxa belonging to individual taxonomy classes between the two cavity-monitoring methods. The frequency of occurrence of taxa belonging to birds, mammals and invertebrates (analyzed at the assigned ranks for taxonomy groups) in the tree cavities was compared in the results obtained using both methods. The Wilcoxon signed-rank test was used to compare the proportions of individual taxa recorded using the two monitoring methods. We used the Pearson correlation test to check the relationship between the number of monitored tree cavities and the recorded species richness. All calculations were performed in the R (ver. 4.5.3) environment using the following packages: dunn.test, ggpubr, and ggplot2 [45].

## 3. Results

### 3.1. External Monitoring of Cavity Use

Camera-trap recordings revealed the presence of 34 animal species, of which 85% were birds, 12% mammals, and 3% insects (Table 1). The Black Woodpecker was the most frequently recorded species and the only species detected at more than 50% of the monitored cavities. Other species with occurrence rates exceeding 30% included the Stock Dove (*Columba oenas*), Great Tit (*Parus major*), Eurasian Pygmy Owl (*Glaucidium passerinum*), and Great Spotted Woodpecker (*Dendrocopos major*). The Common Goldeneye (*Bucephala clangula*), Eurasian Nuthatch (*Sitta europaea*), and Beech Marten (*Martes foina*) were recorded at more than 20% of the monitored cavities (Figure 1). The remaining species were recorded at fewer than 20% of the cavities (Table 1). Differences in the frequency of cavity use among birds and mammals were statistically significant (Wilcox test: V = 1151, *p* < 0.001). Birds were recorded more often than mammals by camera traps. 

Based on the frequency and characteristics of observations, 42% of the identified taxa were considered to use tree cavities for breeding; this group included birds and the Red Squirrel. Another 36% of the taxa were classified as occasional users, as they were recorded only once or twice near a cavity. A smaller proportion of species used cavities as shelters or foraging sites. The Black Woodpecker was classified as a species strongly associated with the cavities it excavates; nevertheless, it was not recorded at approximately one-third of the monitored cavities during camera-trap monitoring (Figure 2).

According to the frequency-based classification of cavity use, 58% of the identified taxa were classified as accidental users, whereas 27% were classified as facultative users. Black Woodpecker cavities were deemed a critical breeding resource for the Stock Dove, Tawny Owl (*Strix aluco*), Common Goldeneye, Great Tit, Eurasian Nuthatch, and Common Redstart (*Phoenicurus phoenicurus*), all of which were recorded in at least 20% of the monitored cavities (Figure 2). Five camera traps did not record any animals, whereas the highest number of taxa recorded by a single camera trap was 14. Camera traps recording fewer than eight taxa were predominant (Figure 3). The Shannon–Wiener diversity index was 1.32.

### 3.2. Internal Monitoring of Cavity Use

A total of 681 Black Woodpecker cavities were inspected using an action camera mounted on a telescopic pole. During these inspections, the presence of at least 33 taxa, including species and higher taxonomic categories, was confirmed. Of these, 52% were birds, 15% mammals, and 33% invertebrates (Table 2, Figure 4). Among the invertebrates, taxa belonging to the families Vespidae, Staphylinidae, Silphidae, and Formicidae, as well as the orders Dermaptera and Lepidoptera, and the class Arachnida, were recorded. The Stock Dove was the most frequently observed species, with nests recorded in 43% of the inspected cavities. Breeding activity of the Black Woodpecker, Common Goldeneye, Tengmalm’s Owl (*Aegolius funereus*), and European Hornet was recorded in more than 10% of the inspected cavities (Table 2, Figure 4).

Other regular, although less frequent, users of Black Woodpecker cavities for breeding included the Common Redstart, Great Tit, Coal Tit (*Periparus ater*), Eurasian Pygmy Owl, Red Squirrel (*Sciurus vulgaris*), Western Honey Bee, and wasps (Vespidae). Other species were recorded breeding only sporadically (Table 2). Invertebrates, including beetles, woodlice, ants, and arachnids, also regularly used Black Woodpecker cavities for feeding and foraging. Differences in the frequency of cavity use among taxonomic classes were not statistically significant (Kruskal–Wallis test: χ^2^ = 2.29, *p* = 0.32).

Many of the recorded organisms could not be identified to the species level; therefore, the actual number of taxa using the cavities was likely higher. Most bird species recorded inside the cavities used them for breeding, as did the recorded mammals. Bats were also recorded using Black Woodpecker cavities for reproduction. We found colonies consisting of juveniles and adults in tree cavities. Among invertebrates, Hymenoptera were recorded breeding inside the cavities, whereas the remaining taxa primarily used cavities as foraging sites or shelters (Table 2). The Shannon–Wiener diversity index calculated for the recorded species and higher taxonomic units was 0.81.

Between survey seasons, there were differences in the size and structure of the populations of animals inhabiting tree cavities. In 2022, a survey of 29 tree cavities revealed the presence of 12 species, and the Shannon index was 0.736. In 2023, 19 species were recorded in 140 tree cavities, and the Shannon index was 0.795. In 2024, a survey of 168 tree cavities revealed 20 species, and the diversity index was 0.765. In 2025 and 2026, we recorded the presence of 23 taxa in 186 and 148 tree cavities, respectively. The Shannon diversity index was 0.936 in 2025 and 0.983 in 2026. The results of the Pearson correlation test indicate a positive relationship between the number of tree cavities surveyed and the recorded species richness (R = 0.92, *p* = 0.03, t = 3.98, df = 3).

### 3.3. Total Diversity

In total, 50 taxa were recorded, including 32 bird species. Among the 32 recorded bird species, fourteen were recorded using both methods, 15 exclusively by camera traps, and three exclusively during internal cavity inspections. Although the two methods produced similar numbers of records, they differed substantially in the composition of the recorded assemblages. Camera traps detected significantly more bird species associated with Black Woodpecker cavities, whereas internal cavity inspections enabled the detection of numerous invertebrate taxa that were not visible in camera-trap recordings.

Six mammal species were recorded in total, of which four were detected by camera traps and five during internal cavity inspections. The results for invertebrates differed markedly between the two methods. Camera traps detected only one species, the European Hornet, whereas internal cavity inspections revealed an additional 10 taxa. The differences in the frequency of occurrence of taxa belonging to birds, mammals, and invertebrates recorded using the two methods were statistically significant (Wilcoxon test: V = 352, *p* = 0.001).

The proportion of cavities in which individual taxa were recorded also differed between the two monitoring methods. The mean proportion of cavities in which a given species was recorded by camera traps was 14.3%, compared with 4.3% for internal cavity inspections. These differences reflect the fundamentally different types of information provided by the two methods and limit direct comparisons of their occurrence estimates. Camera traps generally recorded animals associated with a greater proportion of cavities than did internal inspections. The difference was statistically significant (V = 1032, *p* < 0.001). The results were relatively similar only for the Stock Dove, whereas substantially larger differences were observed for the remaining taxa (Table 3).

## 4. Discussion

### 4.1. Cavity Users Assemblages

Our study in the Augustów Forest demonstrated that Black Woodpecker cavities were important resources for a wide range of forest taxa, including both invertebrates and vertebrates. The use of two complementary monitoring techniques enabled us to collect information on cavity use that would have been difficult to obtain using conventional observation methods. Depending on the monitoring method, 33 and 34 taxa were recorded, respectively. The number of taxa detected by the two methods was similar, which does not support our hypothesis that camera traps would detect a greater number of associated species. When the results of both methods were combined, a total of 50 taxa were recorded. To our knowledge, this represents the largest number of animal taxa documented as using Black Woodpecker cavities within a single forest complex.

The actual number of taxa associated with these cavities is likely higher because bats and several groups of insects and arachnids could not be identified to the species level. More detailed taxonomic investigations, particularly of invertebrates, would probably substantially increase the number of species known to use tree cavities. Furthermore, our study did not cover species using cavities during the entire year, particularly during autumn and winter. Thus, the importance of Black woodpecker cavities as structural resources in the forest ecosystem is likely even greater than indicated by our results. Fungi and other microorganisms inhabiting cavities were also not included in the study and would further increase the diversity of organisms associated with these structures.

Previous studies using cameras installed near tree cavities have generally documented fewer species than were recorded in the Augustów Forest. In eastern Ukraine, Yatsiuk [6] recorded a lower number of species associated with large natural tree cavities. In a beech forest in Germany, Zahner et al. [34] documented 28 vertebrate species, including birds and mammals, using cavities excavated by the Black Woodpecker. By comparison, direct inspections of cavity contents by tree climbing in Sweden documented 14 animal species breeding in Black woodpecker cavities [16].

The number and composition of species recorded in tree cavities may also depend on their geographic distribution. For example, dormice (Gliridae), which use tree cavities as shelters and breeding sites in other parts of Europe [6,12], do not occur in the study area. Kobayashi et al. [46] also demonstrated differences in the assemblages of cavity users depending on stand age. To our knowledge, no published studies have yet compared the number of species detected using cameras positioned inside tree cavities, which limits direct comparisons with our results.

Our study confirmed the multifunctional use of Black Woodpecker cavities by a wide range of animal species. Breeding was one of the most important functions, and was confirmed for at least 14 species, predominantly birds. These species appear to be particularly dependent on the availability of suitable cavities. In addition to the Black Woodpecker itself, the most frequent breeding users included the Stock Dove, Common Goldeneye, Tengmalm’s Owl, European Hornet, and Western Honey Bee. Other breeding species used cavities less frequently, either optionally or only occasionally. These included the Common Redstart, Eurasian Nuthatch, tits, Tawny Owl, Beech Marten, Red Squirrel, bees, and wasps. Single breeding records were obtained for the Goosander, Eurasian Wryneck, Common Starling, Common Blackbird, Common Cuckoo, and some species of bats (Chiroptera). As predicted by our hypothesis, large tree cavities were particularly important for medium-sized birds. However, the frequent use of these cavities by small-bodied species was unexpected and indicates that cavity entrance size alone does not fully determine cavity suitability.

Resting and sheltering represented another important function of the cavities and involved many of the breeding species as well as other taxa. Large cavities provided shelter for animals of different body sizes, including several woodpecker species. The importance of tree cavities as shelters and resting sites has been highlighted in numerous studies [1,4,5,6,8]. Camera-trap recordings documented numerous bird species visiting cavity entrances, although the purpose of individual visits could often not be determined with certainty. These observations were therefore classified as exploratory or visiting behavior and may have represented attempts to locate potential resting or feeding sites.

The presence of predators at cavity entrances was particularly noteworthy. The Beech Marten was the most frequent predatory mammal recorded and has previously been identified as an important predator of cavity users [47,48,49]. It was recorded at approximately 30% of the cavities monitored by camera traps and was the only predatory mammal confirmed to breed in a cavity. The Pine Marten was recorded less frequently and we did not recorded breeding of this species in cavities. These observations suggest that pressure of martens was the most important threat for other cavity users, as also reported in previous studies [6,12,34]. Among raptors, the Tawny Owl was the most frequent cavity-associated species. It both bred in cavities and visited additional cavities. Tengmalm’s Owl used cavities for breeding and food storage and occasionally visited other cavities [7]. Single observations of the Common Buzzard (*Buteo buteo*) and Eurasian Pygmy Owl were also recorded. The Tawny Owl therefore represented an interesting example of a species that both uses cavities as a reproductive resource and may possibly be exposed to predation by martens itself [7], although we do not have any recordings of such incidents. However, we have documented a case of a brood being destroyed by a predator, probably a marten. Overall, several species used cavities for multiple purposes, and the strength of their association with cavities varied considerably. The importance of tree cavities for interspecific interactions has also been emphasized by Gorman [12] and Yatsiuk [6].

### 4.2. Comparing of the Monitoring Methods 

The use of two complementary monitoring techniques [21,22,23,27,36] confirmed the association of Black Woodpecker cavities with a diverse assemblage of 50 animal taxa. However, the information obtained using the two methods differed substantially. The Shannon–Wiener diversity index was higher for the camera-trap’s generally smaller dataset, likely reflecting the higher frequency of occurrence recorded for many species approaching the tree cavities. On the other hand, these indices for both methods were calculated based on different, not entirely comparable samples. Camera traps monitored cavity entrances over extended periods and recorded animals approaching, flying to, or climbing toward the cavity entrance [6,34]. However, they did not allow for cavity use to be determined conclusively in all cases, particularly when reproductive attempts failed or nests were abandoned during incubation. 

An important advantage of camera traps is their ability to collect data continuously over several months or even across multiple seasons. Nevertheless, camera-trap recordings are subject to imperfect detection. Not all animals approaching or leaving cavities were necessarily captured by the camera. Species entering or leaving the cavity rapidly, particularly without pausing at the entrance, could easily escape detection. This was illustrated by recordings in which only the entrance or only the exit of an animal was captured, although both events must have occurred. The results obtained are greatly influenced by the technical specification of the cameras used, the distance from the monitored tree cavity, and how the cameras are positioned [50]. Consequently, the species richness estimated from camera-trap recordings should be regarded as a minimum estimate. Increasing the number of monitored cavities and extending the monitoring period would probably result in the detection of additional species and provide more precise estimates of the strength of associations between individual taxa and Black Woodpecker cavities. Camera traps were also relatively ineffective for detecting invertebrates: the European Hornet was the only invertebrate taxon reliably recorded, consistent with the findings of Yatsiuk [6].

Cameras positioned inside tree cavities provided detailed information on cavity contents at the time of inspection, including the presence of nests, broods, and different stages of reproduction [35]. However, this method provided little information about animals visiting the cavity entrance between inspections. An important practical advantage is that internal inspections can be conducted from the ground and do not require climbing the tree. The method also reduces the time required to inspect individual cavities and may reduce disturbance to cavity occupants. Several dozen cavities can be inspected during a single day, making this technique particularly useful for large-scale surveys.

The two methods should therefore not be considered as alternatives, and their results cannot be directly interpreted as measures of the same ecological process. Instead, they provide complementary information on cavity use. Camera traps provide information on long-term activity at cavity entrances, including repeated visits and the relative frequency of occurrence of different species. Internal inspections, in contrast, provide a direct assessment of cavity contents and allow for reproductive activity to be documented with considerably greater certainty.

The internal view therefore provides particularly valuable information on reproduction and enables the identification of species that are strongly associated with cavities but remain hidden from external observation. External camera traps are highly useful for monitoring vertebrates and documenting long-term activity but provide very limited information on invertebrates inhabiting cavities. Detailed inspection of cavity interiors is therefore necessary to detect a substantial proportion of the cavity-associated fauna. Both methods provide considerably more information than observations conducted exclusively from the ground and can reduce the time and effort required for cavity monitoring. However, their implementation requires specialized equipment and additional logistical resources.

### 4.3. Seasonal Variability and Limitations of Obtained Results

The seasonal variations observed in the results obtained from the mast-mounted camera indicate the variability in results obtained during a single research season. Excluding 2022, when the sample size was much smaller, the results for 2023–2026 ranged from 19 to 23 recorded taxa, and the diversity index varied from 0.73 to 0.98. During those years, an average of 160 tree cavities (140–186) were surveyed. Meanwhile, with a sample size of 39 tree cavities in 2022, 12 species were recorded. This shows that sample size increases the recorded species richness and the value of the diversity index. However, due to the limited number of seasons in only 5, this relationship is not very precise and requires a larger sample size over a longer period of time to verify the obtained result.

The results presented here do not constitute an exhaustive list of animal species present in black woodpecker tree cavities. It is important to bear in mind certain limitations resulting from the size of the study sample. While the sample size for the mast-based survey allowed for a fairly comprehensive picture of the breeding community structure in the tree cavities, the number of camera traps used did not allow for such an assessment. In addition, the camera traps were not distributed evenly; most of them were installed in the national park. It would likely be beneficial to expand the scale and duration of the camera trap surveys; enabling the monitoring of 30–40 tree cavities per year over several breeding seasons would yield more comprehensive results. Additionally, it would be valuable to assess cavity use on an annual cycle and the variability in the use of the same cavities across successive breeding seasons. Due to limitations resulting from the large disparity between the number of tree cavities monitored by each method and the varying lengths of the study periods, we did not opt for an expanded analytical approach to the results obtained through both methods.

In our assessment, installing camera traps on a different tree—especially before the breeding season—did not cause the animals to avoid the tree cavities. In several cases, it was clearly evident that birds (titmice, flycatchers, and tree sparrows) were interested and were flying from the tree cavities to the camera traps. No similar behavior was recorded among mammals. 

The second research method, involving the presence of a camera inside the tree cavity, may have had a greater potential impact. However, despite this concern, we did not observe any noticeable impact. The studied species did not disappear during subsequent inspections. Birds and mammals continued the broods they had started. We also frequently observed insects during subsequent visits. Hence, our assessment is that the conducted research did not cause disturbances in the animals’ lives nor did it affect the results of the cavity occupancy assessments.

Furthermore, it should be emphasized that these results were obtained in a single study area with a relatively high species diversity, using specific equipment and methodology. Results obtained in other locations may vary; the use of higher-quality cameras in the future may make it possible to capture and identify a wider range of invertebrates and alter the structure of the community.

## 5. Conclusions

Our study confirmed the importance of large tree cavities for maintaining forest biodiversity. The results also indicate that Black Woodpecker cavities support a diverse assemblage of organisms and may facilitate numerous, and in some cases poorly understood, ecological interactions among cavity users. Not all species can use every cavity, and cavity characteristics are likely to influence their suitability for different users. Further research should therefore focus on identifying the structural characteristics of cavities preferred by particular taxa, which could improve the management and conservation of cavity-bearing trees and their surrounding habitats.

More detailed investigations of invertebrates are likely to substantially increase our knowledge of the diversity of cavity-associated fauna. Camera traps positioned near cavity entrances may also detect additional insect taxa, particularly with improvements in camera sensitivity and monitoring duration. The importance of tree cavities for different groups of Hymenoptera and their preferences for particular cavity characteristics and locations warrant further investigation, based on currently available technologies as well as new ones that are emerging. Future studies could also benefit from cameras positioned inside cavities for longer periods, allowing for continuous monitoring of cavity contents and providing additional information on breeding activity, interactions among cavity users, and changes in cavity occupancy over time. It should be noted that factors of seasonality and cavity location may influence usage by other animals but that this requires further analysis based on the data obtained in the article.

## Figures and Tables

**Figure 1:**
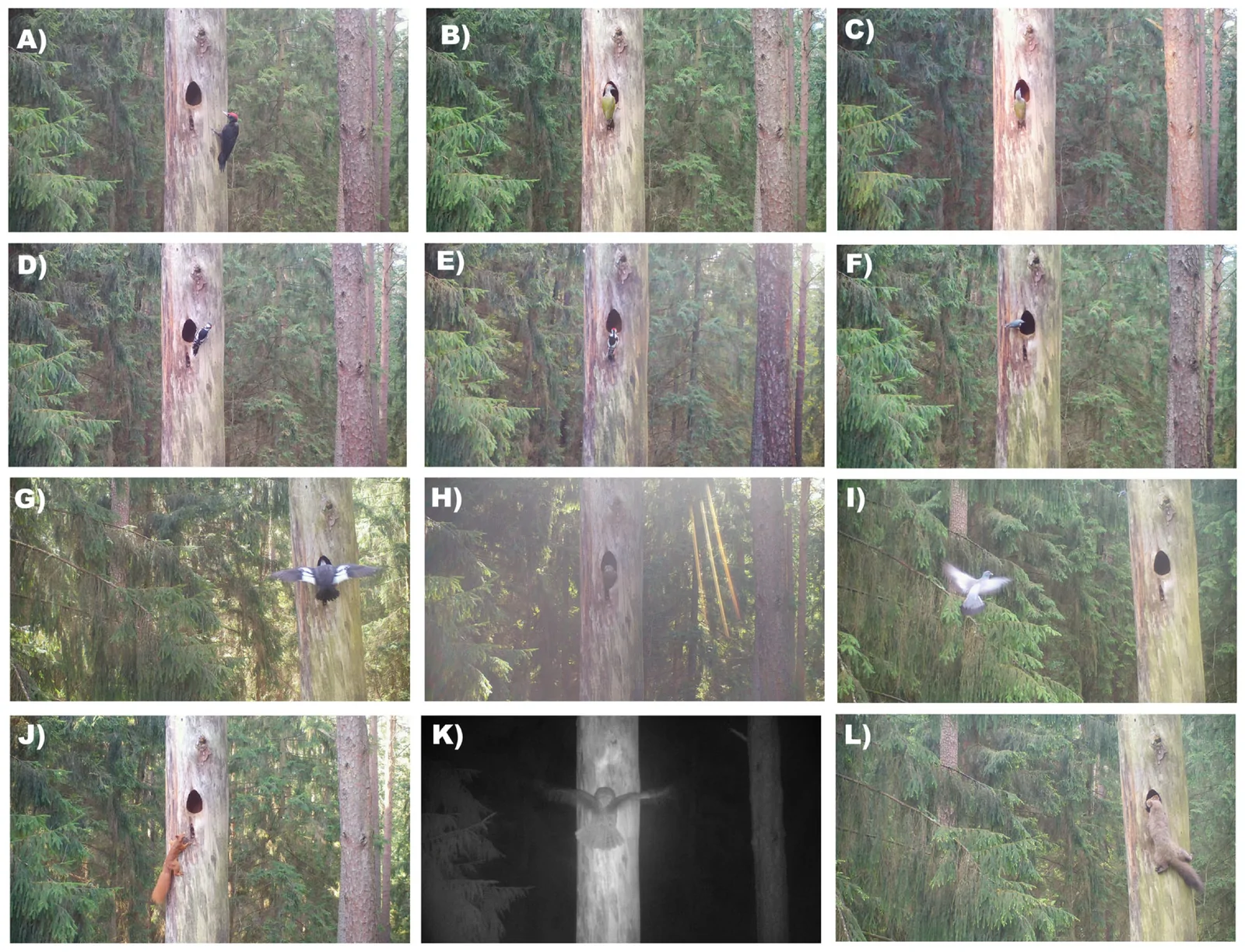
Animals recorded by camera trap near the tree cavity with the highest species richness ((**A**)—Black woodpecker, (**B**)—Grey-headed woodpecker, (**C**)—Green woodpecker, (**D**)—Great spotted woodpecker, (**E**)—Middle spotted woodpecker, (**F**)—Eurasian nuthatch, (**G**)—Common goldeneye, (**H**)—Eurasian pygmy owl, (**I**)—Stock dove, (**J**)—Red squirrel, (**K**)—Tawny owl, (**L**)—Beech marten).

**Figure 2:**
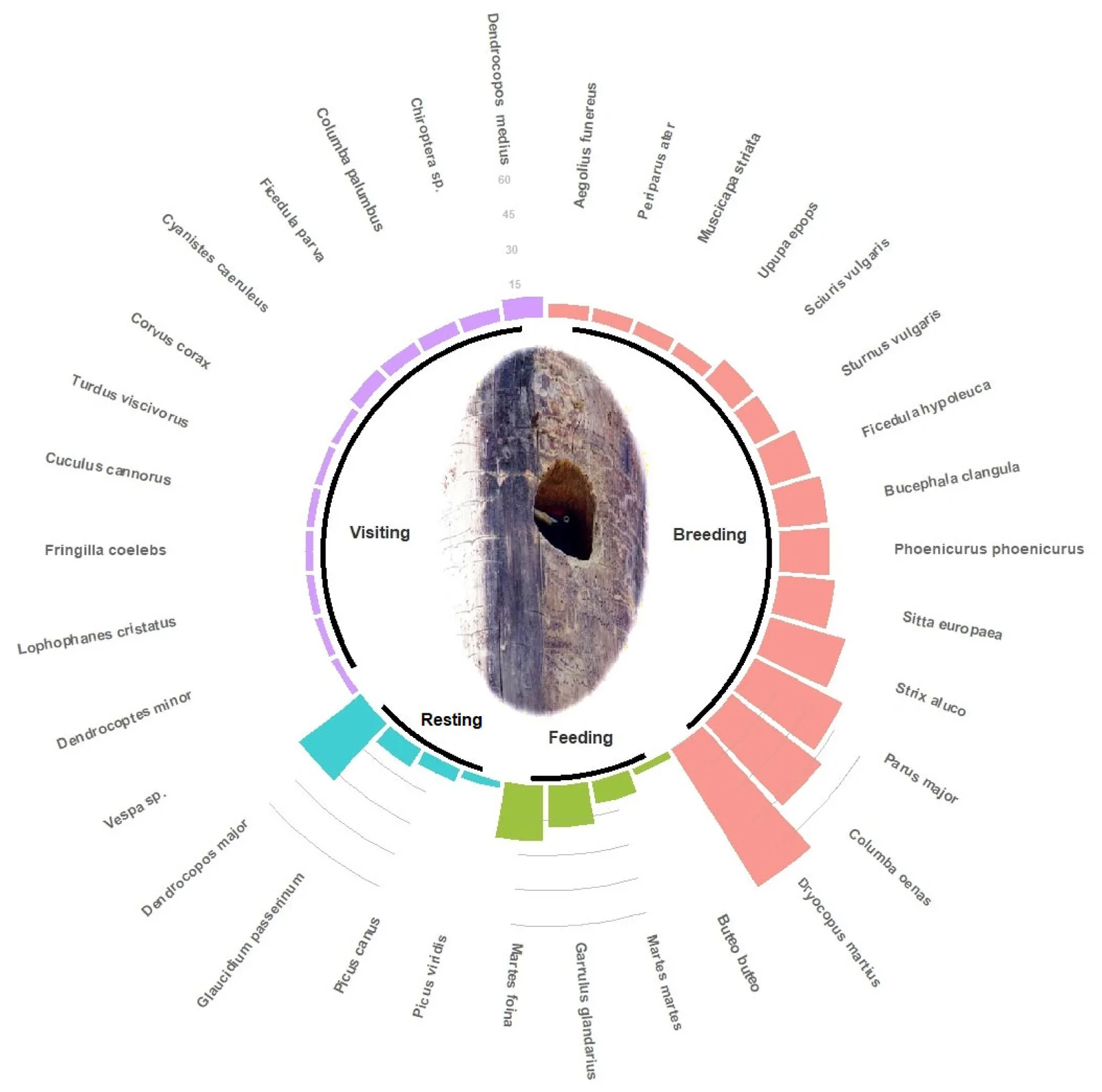
The type and strength of links with Black Woodpecker cavities for species detected by camera traps.

**Figure 3:**
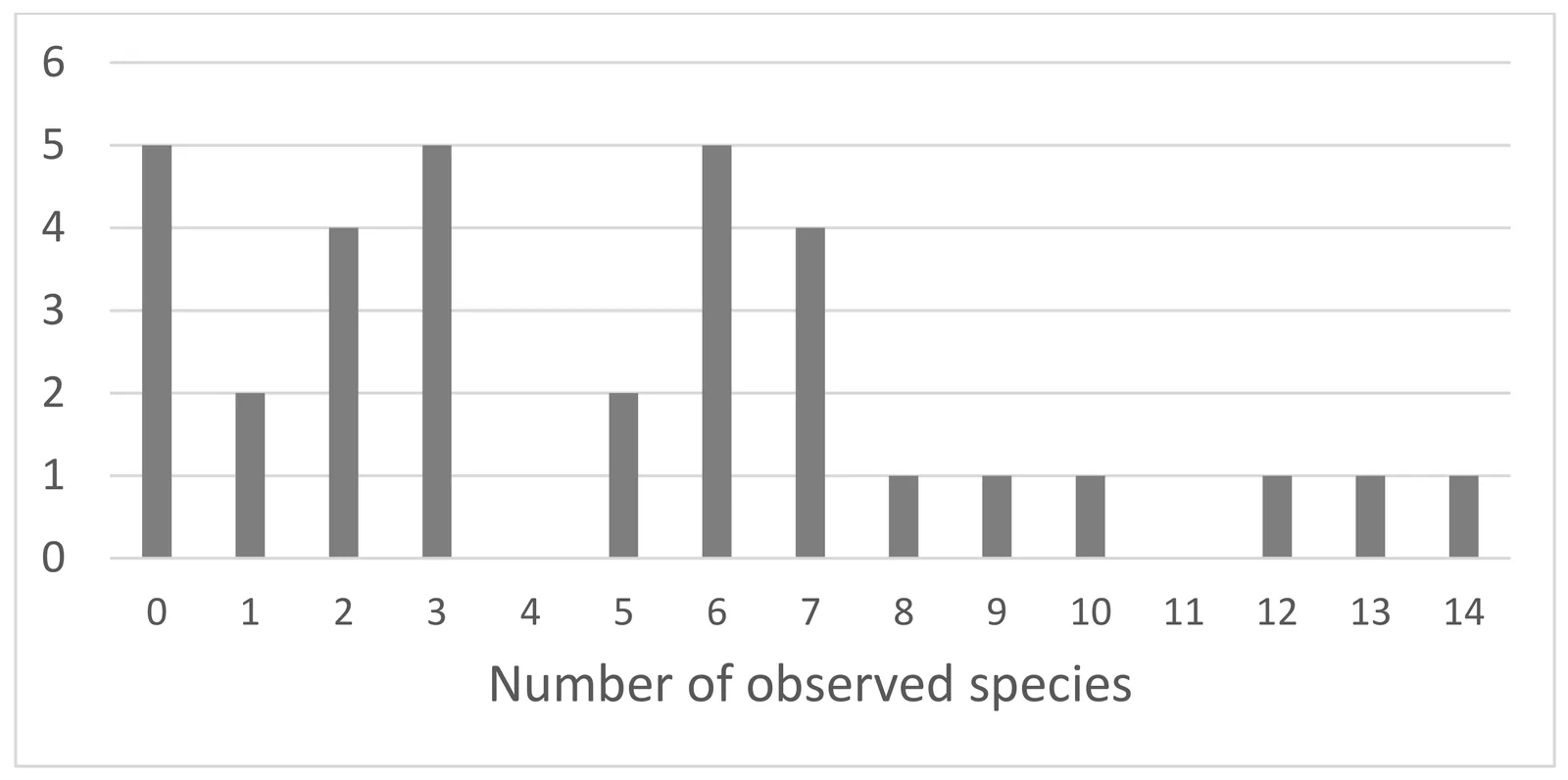
Number of camera traps ranked by the number of species observed.

**Figure 4:**
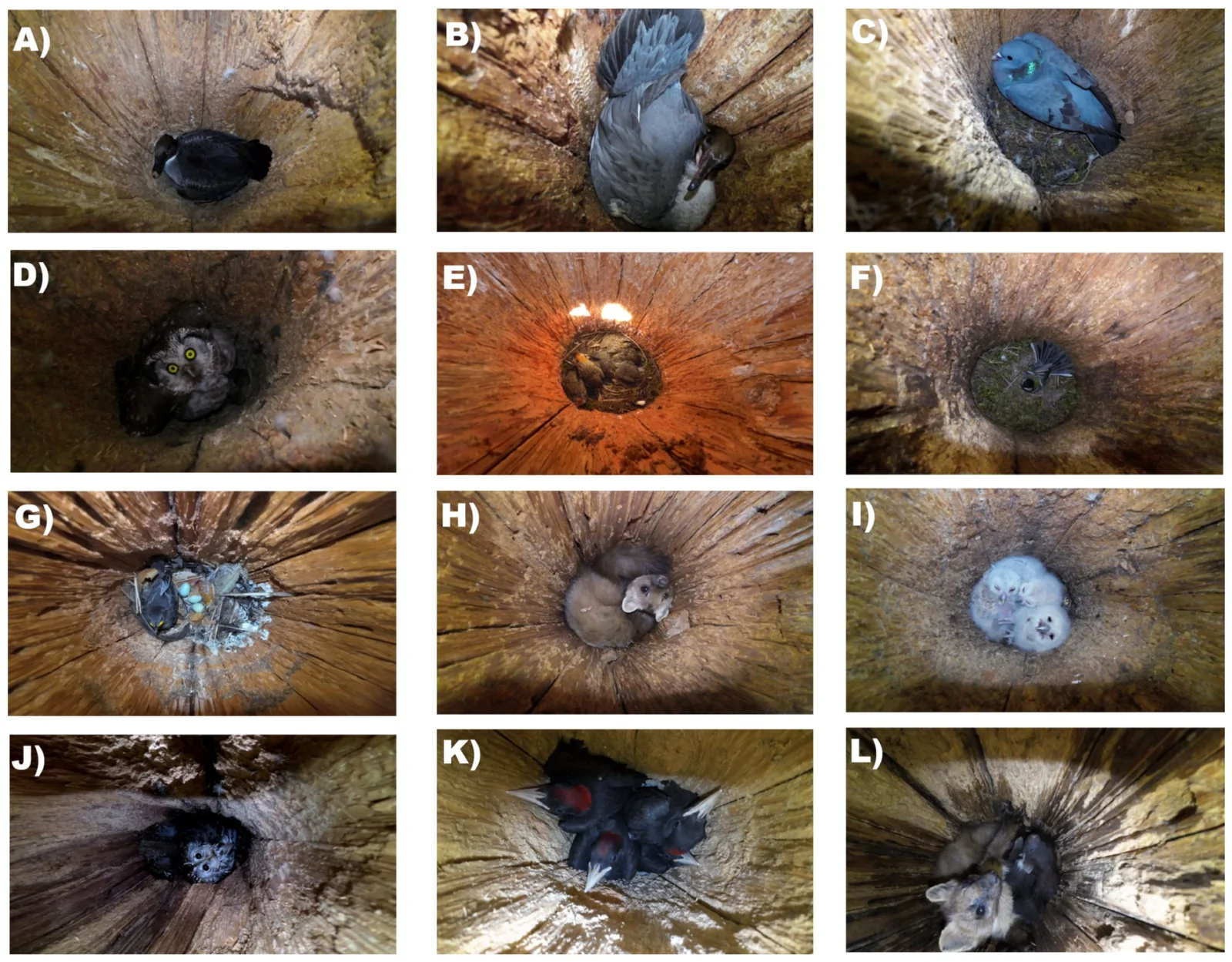
Examples of species recorded in the Black woodpecker cavities ((**A**)—Common goldeneye, (**B**)—Goosander, (**C**)—Stock dove, (**D**)—Tengmalm’s Owl, (**E**)—Young Common redstarts, (**F**)—Great tit, (**G**)—young Common starling, (**H**)—Beech marten, (**I**)—Young Tawny owls, (**J**)—Tawny owl, (**K**)—Young black woodpeckers, (**L**)—Beech marten with offspring).

**Table 1:** The list of species recognized in data collected by camera traps near black woodpecker cavities.

Scientific Name	English Name	Type of Using	Type of Correlation	Prevalence Rate (%)
*Dryocopus martius*	Black woodpecker	Breeding	Keystone	70
*Dendrocopos major*	Great spotted woodpecker	Resting	Facultative	33
*Dendrocopos medius*	Middle spotted woodpecker	Visiting	Accidental	9
*Dendrocoptes minor*	Lesser spotted woodpecker	Visiting	Accidental	3
*Picus canus*	Grey-headed woodpecker	Resting	Accidental	6
*Picus viridis*	Green woodpecker	Resting	Accidental	3
*Columba oenas*	Stock dove	Breeding	Keystone	48
*Bucephala clangula*	Common goldeneye	Breeding	Keystone	21
*Strix aluco*	Tawny owl	Breeding	Keystone	33
*Aegolius funereus*	Tengmalm’s owl	Breeding	Accidental	6
*Glaucidium passerinum*	Eurasian pygmy owl	Resting	Accidental	9
*Parus major*	Great tit	Breeding	Keystone	42
*Periparus ater*	Coal tit	Breeding	Accidental	6
*Cyanistes caeruleus*	Blue tit	Visiting	Accidental	6
*Lophophanes cristatus*	Crested tit	Visiting	Accidental	3
*Sitta europaea*	Eurasian nuthatch	Breeding	Facultative	24
*Phoenicurus phoenicurus*	Common redstart	Breeding	Facultative	21
*Ficedula hypoleuca*	European pied flycatcher	Breeding	Facultative	18
*Ficedula parva*	Red-breasted flycatcher	Visiting	Accidental	6
*Muscicapa striata*	Spotted flycatcher	Breeding	Accidental	6
*Sturnus vulgaris*	Common starling	Breeding	Facultative	12
*Fringilla coelebs*	Eurasian chaffinch	Visiting	Accidental	3
*Columba palumbus*	Wood pigeon	Visiting	Accidental	6
*Cuculus cannorus*	Common cuckoo	Visiting	Accidental	3
*Turdus viscivorus*	Mistle thrush	Visiting	Accidental	3
*Corvus corax*	Common raven	Visiting	Accidental	3
*Buteo buteo*	Common buzzard	Feeding	Accidental	3
*Garrulus glandarius*	Eurasian jay	Feeding	Facultative	18
*Upupa epops*	Eurasian hoopoe	Breeding	Accidental	6
*Martes foina*	Beech marten	Feeding	Facultative	27
*Martes martes*	Pine marten	Feeding	Facultative	9
*Sciuris vulgaris*	Red squirrel	Breeding	Facultative	12
*Chiroptera*	Bat unidentified	Visiting	Accidental	6
*Vespa crabro*	European hornet	Visiting	Accidental	3

**Table 2:** List of species identified based on data collected by camera inside Black woodpecker cavities.

Scientific Name	English Name	Type of Using	Type of Correlation	Prevalence Rate (%)
*Dryocopus martius*	Black woodpecker	Breeding	Keystone	18
*Dendrocopos major*	Great spotted woodpecker	Resting	Accidental	0.3
*Picus viridis*	Green woodpecker	Resting	Accidental	0.3
*Jynx torquilla*	Eurasian wryneck	Breeding	Accidental	0.2
*Columba oenas*	Stock dove	Breeding	Keystone	44.1
*Bucephala clangula*	Common goldeneye	Breeding	Keystone	11.8
*Mergus merganser*	Goosander	Breeding	Accidental	0.8
*Strix aluco*	Tawny owl	Breeding	Facultative	0.9
*Aegolius funereus*	Tengmalm’s owl	Breeding	Keystone	15
*Glaucidium passerinum*	Eurasian pygmy owl	Breeding	Accidental	1.1
*Phoenicurus phoenicurus*	Common redstart	Breeding	Facultative	5.8
*Parus major*	Great tit	Breeding	Facultative	3.6
*Periparus ater*	Coal tit	Breeding	Accidental	0.9
*Tudus merula*	Common blackbird	Breeding	Accidental	0.2
*Sitta europaea*	Eurasian nuthatch	Breeding	Accidental	2.7
*Cuculus cannorus*	European cuckoo	Breeding	Accidental	0.2
*Sturnus vulgaris*	Common starling	Breeding	Accidental	1.6
*Martes foina*	Beech marten	Breeding	Accidental	1.1
*Sciuris vulgaris*	Red squirrel	Breeding	Accidental	1.7
*Pipistrellus nathusii*	Nathusius’s pipistrelle	Breeding	Accidental	0.8
*Nyctalus leisleri*	Lesser noctule	Breeding	Accidental	0.1
*Cnephaeus serotinus*	Serotine bat	Resting	Accidental	0.1
*Vespa crabro*	European hornet	Breeding	Keystone	11.6
*Apis mellifera*	Western honey bee	Breeding	Keystone	3.1
*Vespula* sp.	Wasp unidentified	Breeding	Accidental	2.0
*Bombus* sp.	Bumblebee unidentified	Breeding	Accidental	0.3
*Velleius dilatatus*	Hornet rove beetle	Feeding	Accidental	2.3
*Oiceoptoma thoracicum*	Red-breasted carrion beetle	Feeding	Accidental	0.4
*Nicrophorus vespilloides*	Lesser vespillo burying beetle	Feeding	Accidental	0.6
*Antidae* sp.	Ant unidentified	Feeding	Accidental	6.6
*Arachnida* sp.	Arachnid unidentified	Resting	Accidental	3.4
*Heterocera* sp.	Moth unidentified	Resting	Accidental	0.5
*Dermaptera* sp.	Earwig unidentified	Feeding	Accidental	0.3

**Table 3:** A complete list of species associated with Black Woodpecker cavities, using both methods (camera traps and pole-mounted cameras).

Number	Scientific Name	English Name	Camera Trap (%)	Pole-Mounted Camera (%)	Type of Using
1	*Dryocopus martius*	Black woodpecker	70	18	Breeding
2	*Dendrocopos major*	Great spotted woodpecker	33	0.3	Resting
3	*Dendrocopos medius*	Middle spotted woodpecker	9	0	Resting
4	*Dendrocoptes minor*	Lesser spotted woodpecker	3	0	Resting
5	*Picus canus*	Grey-headed woodpecker	6	0	Resting
6	*Picus viridis*	Green woodpecker	3	0.3	Resting
7	*Jynx torquilla*	Eurasian wryneck	0	0.2	Breeding
8	*Columba oenas*	Stock dove	48	44.1	Breeding
9	*Bucephala clangula*	Common goldeneye	21	11.8	Breeding
10	*Mergus merganser*	Goosander	0	0.8	Breeding
11	*Strix aluco*	Tawny owl	33	0.9	Breeding
12	*Aegolius funereus*	Tengmalm’s owl	6	15	Breeding
13	*Glaucidium passerinum*	Eurasian pygmy owl	9	1.2	Resting
14	*Parus major*	Great tit	42	3.6	Breeding
15	*Periparus ater*	Coal tit	6	0.9	Breeding
16	*Cyanistes caeruleus*	Blue tit	6	0	Visiting
17	*Lophophanes cristatus*	Crested tit	3	0	Visiting
18	*Sitta europaea*	Eurasian nuthatch	24	2.7	Breeding
19	*Phoenicurus phoenicurus*	Common redstart	21	5.8	Breeding
20	*Ficedula hypoleuca*	Pied flycatcher	18	0	Breeding
21	*Ficedula parva*	Red-breasted flycatcher	6	0	Visiting
22	*Muscicapa striata*	Spotted flycatcher	6	0	Breeding
23	*Sturnus vulgaris*	Common starling	12	1.6	Breeding
24	*Fringilla coelebs*	Eurasian haffinch	3	0	Visiting
25	*Columba palumbus*	Wood pigeon	6	0	Visiting
26	*Cuculus cannorus*	Common cuckoo	3	0.2	Breeding
27	*Turdus merula*	Common blackbird	0	0.2	Breeding
28	*Turdus viscivorus*	Mistle thrush	3	0	Visiting
29	*Corvus corax*	Common raven	3	0	Visiting
30	*Buteo buteo*	Common buzzard	3	0	Feeding
31	*Garrulus glandarius*	Eurasian jay	18	0	Feeding
32	*Upupa epops*	Eurasian hoopoe	6	0	Breeding
33	*Martes foina*	Beech marten	27	1.1	Breeding
34	*Martes martes*	Pine marten	9	0	Feeding
35	*Sciuris vulgaris*	Red squirrel	12	1.7	Breeding
36	*Chiroptera* sp.	Bat unidentified	6	0	Feeding
37	*Pipistrellus nathusii*	Nathusius’s pipistrelle	0	0.8	Breeding
38	*Nyctalus leisleri*	Lesser noctule	0	0.1	Breeding
39	*Cnephaeus serotinus*	Serotine bat	0	0.1	Resting
40	*Vespa crabro*	European hornet	3	11.6	Breeding
41	*Apis mellifera*	Western honey bee	0	3.1	Breeding
42	*Vespula* sp.	Wasp unidentified	0	2.0	Breeding
43	*Bombus* sp.	Bumblebee unidentified	0	0.3	Breeding
44	*Velleius dilatatus*	Hornet rove beetle	0	2.4	Feeding
45	*Oiceoptoma thoracicum*	Red-breasted carrion beetle	0	0.4	Feeding
46	*Nicrophorus vespilloides*	Lesser vespillo burying beetle	0	0.6	Feeding
47	*Antidae* sp.	Ant unidentified	0	6.6	Feeding
48	*Arachnida* sp.	Arachnid unidentified	0	3.4	Resting
49	*Heterocera* sp.	Moth unidentified	0	0.5	Resting
50	*Dermaptera* sp.	Earwig unidentified	0	0.3	Feeding

## Data Availability

The raw data supporting the conclusions of this article will be made available by the authors on request.

## Supplementary Materials

The following supporting information can be downloaded at: https://www.mdpi.com/article/10.3390/biology15191687/s1, Table S1: Data used in the preparation of this article—the abundance of individual species recorded in individual tree cavities using camera traps and in specific years of the study using pole-mounted cameras.
